# Factors Affecting Patients’ Atttendance for Periodontal Follow-up Visits after Crown Lengthening Surgery

**DOI:** 10.3290/j.ohpd.b5569483

**Published:** 2024-07-11

**Authors:** Sumaiah A. Ajlan, Shoag M. Hummady, Alanoud A. Salam, Arwa A. Talakey, Nahid Y. Ashri, Amani A. Mirdad, Marwa Y. Shaheen, Amani M. Basudan, Mansour H. Alaskar, Hani S. AlMoharib, Fatemah Al-Ahmari

**Affiliations:** a Assistant Professor, Department of Periodontics and Community Dentistry, College of Dentistry, King Saud University, Riyadh, Saudi Arabia. Planned the research, participated in data collection, drafted the manuscript, read and approved the final manuscript.; b Intern, College of Dentistry, King Saud University, Riyadh, Saudi Arabia. Reviewed the files for collection of demographic data, read and approved the final manuscript.; c Assistant Professor, Department of Periodontics and Community Dentistry, College of Dentistry, King Saud University, Riyadh, Saudi Arabia. Reviewed the study design, performed the statistical analysis, read and approved the final manuscript.; d Professor, Department of Periodontics and Community Dentistry, College of Dentistry, King Saud University, Riyadh, Saudi Arabia. Reviewed the paper, helped in writing the discussion, read and approved the final manuscript.; e Assistant Professor, Department of Periodontics and Community Dentistry, College of Dentistry, King Saud University, Riyadh, Saudi Arabia. Called the patients, registered their response and arranged their booking, read and approved the final manuscript.; f Assistant Professor, Department of Periodontics and Community Dentistry, College of Dentistry, King Saud University, Riyadh, Saudi Arabia. Searched the files, reviewed the progress notes for initial identification of candidate patients, read and approved the final manuscript.; g Professor, Department of Periodontics and Community Dentistry, College of Dentistry, King Saud University, Riyadh, Saudi Arabia. Called the patients, registered their response and arranged their booking, read and approved the final manuscript.; h Associate Professor, Department of Periodontics and Community Dentistry, College of Dentistry, King Saud University, Riyadh, Saudi Arabia. Searched the files, reviewed the progress notes for initial identification of candidate patients, read and approved the final manuscript.

**Keywords:** adherence, compliance, crown lengthening surgery, periodontal maintenance

## Abstract

**Purpose:**

To assess adherence to follow-up maintenance visits among patients who had previously undergone crown-lengthening surgery and investigate the different factors impacting their compliance.

**Materials and Methods:**

A total of 314 patients were identified for follow-up appointments. Based on their responses, participants were categorised into four groups: attendees, non-attendees, refusals, and unreachable. Furthermore, data on sociodemographic factors (age, sex, nationality, marital status, education, occupation, and residential area), medical history, dental history (including missing teeth, implants, or orthodontic treatment history), and past appointment attendance (average yearly appointments, missed appointment percentage, and last appointment date) were collected and analysed to understand their influence on patient compliance.

**Results:**

In a sample of 314 patients, 102 (32.5%) attended the appointments successfully. Improved attendance rates were significantly associated with being female, Saudi Arabian, married, and employed (p < 0.05). Moreover, patients with a high frequency of annual appointments and a recent history of appointments exhibited better compliance. None of the analysed dental factors affected the attendance rates.

**Conclusion:**

About one-third of patients who had undergone crown lengthening surgery were compliant with the follow-up visits. Different factors influenced this compliance pattern to varying extents, with more efforts needed to enhance patients’ commitment to these visits.

Scheduling hospital appointments is essential to ensure that patients receive necessary care. Further, patients should attend these appointments to optimise the time and resources needed to manage the patient list effectively.^59^ Dental care is vital in improving oral health across all patient categories. Typically, various types of appointments are available, including consultations, examinations, emergency care, regular treatment visits, and follow-up/recall appointments.^17^^,^^37^^,^^54^

Compliance, previously defined as “the extent to which a person’s behavior aligns with medical or health advice”,^21^^,^^26^ is now commonly referred to as adherence.^48^ Adherence is defined as “the degree to which a person’s behavior aligns with the recommendations of a healthcare provider” and encompasses the patient’s commitment to illness, treatment, and the therapist.^30^^,^^37^^,^^48^

In the Middle East, a few studies have assessed patient compliance with medical appointments, revealing that numerous patients do not adhere to them.^2^^,^^3^^,^^39^^,^^55^ This noncompliance rate generally surpasses the international norms.^6^ A Saudi Arabian study found variances in attendance rates linked to the severity and type of medical discipline, noting greater compliance under serious or painful conditions.^6^ Consequently, dental appointments are often viewed as less critical, leading to lower patient attendance.^6^

In our country, documentation on adherence to dental appointments and follow-up visits is scarce. Nazir reported that only approximately 19% of male Saudi adolescents routinely visit dentists, half of whom suffer from dental caries, tooth sensitivity, or pain.^38^ Awartani found that 12–15% of appointments were missed at a university-based institution,^8^ and Shabbir et al^50^ reported that 58.1% of scheduled visits were not attended at an eastern province military hospital in Saudi Arabia.^50^

Not attending dental appointments can have several negative effects. At the patient level, these absences can disrupt treatment continuity, often leading to adjustments in the initially proposed treatment plan.^25^ In a broader community context, missed appointments result in unused slots and delayed appointments for the next patient.^50^ This increase in waiting lists complicates the necessary treatments and has additional financial implications.^17^^,^^29^^,^^50^

Therefore, factors affecting patient attendance patterns have been extensively studied.^17^^,^^22^^,^^29^ These can be categorised into varying contextual and individual elements,^30^ including sociodemographic factors (age, educational level, and distance from the appointment center^59^), psychological factors (past experiences and dental fear^30^^,^^38^) and health literacy (understanding of treatment plan details and importance of visits).^13^^,^^29^^,^^32^

For instance, Al Barakati^5^ found that factors such as older age and low-to-middle socioeconomic background were associated with appointment-breaking behavior among patients at King Saud University dental clinics.^5^

Dental recall or maintenance appointment is the final and crucial stage in a dental treatment plan.^47^ This phase is important for maintaining the results of previous procedures, preventing disease recurrence, diagnosing diseases early, and treating complications. However, patient compliance with these recall visits is often poorer than with visits for pain management or emergencies.^37^^,^^59^

In periodontology, regular maintenance visits may be as crucial as the treatment itself for preventing further tissue breakdown.^10^^,^^20^^,^^36^^,^^47^ Factors such as disease severity, periodontal prognosis, and the type of therapy can also influence a patient’s adherence to maintenance appointments.^60^ For example, patients who have undergone surgical periodontal therapy are often diligent in attending follow-up dental appointments.^11^^,^^52^^,^^60^ This can be attributed to greater awareness of the complexity of the treatment plan and the desire to avoid a second operation.^37^

Crown-lengthening is a common periodontal surgery^31^ often performed to treat compromised teeth. It has several indications, which includes: gaining access to sound tooth margins for carious or broken teeth,^58^ increasing the crown length, improving the retention of the restoration,^16^^,^^44^ avoiding biologic width violation, ^22^^,^^23^ and improving the esthetic appearance of short anterior crowns.^31^ Patient compliance with supportive therapy to maintain these teeth is essential, and includes adequate plaque control and compliance with recall appointments.^58^ In fact, those patients are expected to show higher compliance rates as they initially were highly motivated to keep their teeth and were keen to undergo several procedures and attend multiple appointments to avoid extraction. However, up to the best of our knowledge no study has directly evaluated the compliance rate of this group.

This study aimed to assess compliance with follow-up periodontal maintenance visits among a group of patients who had previously undergone crown-lengthening surgeries and examine the impact of various factors on their attendance rates.

## MATERIALS AND METHODS

### Ethical Approval

The research proposal was reviewed and approved by the Institutional Review Board of King Saud University Medical City, Riyadh, Saudi Arabia (Res Project No. E-20-4768) after establishing the conditions to ensure patient confidentiality. All patients included have informed consents signed in the file allowing the utilisation of their data for educational and research purposes.

### Design and Setting

This is an observational, cross-sectional study that utilised data from another ongoing study evaluating the long-term survival of teeth after crown-lengthening surgeries (publication in progress). Briefly, patients who underwent crown-lengthening surgery at the College of Dentistry, King Saud University, Riyadh, Saudi Arabia, between 2013 and 2022 were included in this study. Those patients were called and invited to attend a follow up appointment. Patient responses were recorded and correlated with their data previously documented in their files.

### Initial Patient Identification

Our dental college uses an academic dental file system (SALUD; Dentsply,Sirona). This electronic software was searched to locate all patient files that mentioned “crown lengthening” between 2013 and 2022. We only included patients who underwent flap surgeries (excluding gingivectomies) performed more than 12 months previously, with files containing sufficient details about the procedure, and with a signed informed consent. The search identified approximately 314 patients.

### Contacting Patients 

From December 2022 to December 2023, we contacted potential patients via the phone numbers they provided and invited them to participate in a study evaluating the success rate of teeth treated with crown lengthening. We requested that they attend a follow-up examination focused on these teeth. We also offered them regular periodontal recall maintenance services, which comprised holistic dental and periodontal evaluations, potential radiographic examinations, thorough scaling/prophylaxis, and topical fluoride application. If additional treatment was required, the patients were referred to the appropriate department within our institution. Those who were not immediately forthcoming made several attempts at contact and often gave them another opportunity to participate when suitable. The participants’ responses were divided into the following categories:

- Those who agreed to participate and came (attendees).
- Those who agreed to participate but failed to attend (non-attendees).
- Those who refused because they were uninterested (refusals).
- Those who could not be contacted for different reasons (no answer, changed contact, left the area, or unreachable).

### Factors Affecting Patients Atttendance Styles

### Demographic and socioeconomic factors

Basic patient demographics and socioeconomic data were extracted from the files and linked to attitudes toward participation. These characteristics included age, sex, nationality, marital status, household size, educational level, occupation, and proximity to college (classified as short or long distance, < 10 km or > 10 km from the college, respectively).

### Medical history

The documented medical history of the patients was examined and classified using the American Society of Anesthesiologists’ (ASA) classification of physical system.^7^ Subsequently, the potential influence of medical condition on patients’ willingness to attend appointments and the overall compliance rate were assessed.

### Dental history

Preliminary dental histories were obtained by examining patient radiographs, focusing on several key factors:

1. Number of missing teeth (including extracted or hopeless teeth, excluding third molars and those congenitally missing or extracted for orthodontic reasons, where space is closed).

2. History of replacement of missing teeth,

 A: by fixed dental prosthesis (pontic).

 B. by dental implant.

3. History of orthodontic treatment.

We tested for any possible association between those factors and patient attendance status.

### Appointment history

Initially, the total number of patient appointments at the college was tallied. The duration of these appointments in years was ascertained, enabling us to calculate the patients’ annual visit frequencies.

The number of missed appointments, including those not attended, rescheduled, or canceled, was identified. This number was divided by the total number of scheduled appointments to calculate the percentage of missed appointments. Subsequently, the patients were categorised based on this percentage (modified from Novaes et al^41^ and Miyamoto et al^35^).

- Compliant if they missed < 30% of their regular previous appointments.
- Erratic if they missed 30%–60% of their regular previous appointments.
- Non-compliant if they missed > 60% of their regular previous appointments.

The date of the penultimate college appointment before the follow-up was identified, and its numerical value was analysed.^14^^,^^33^ This evaluation was used to classify patients into active (last appointment occurred less than 3 years ago), inactive (last appointment was more than 5 years ago), and moderately active (last appointment was between 3 and 5 years ago) categories.

The association between these factors and patient attendance at the visit was also evaluated.

### Statistical Analysis

We used SPSS version 26 (IBM; Armonk, NY, USA) to analyse the data. Categorical variables are presented as frequencies and percentages, while numerical variables are described using means and standard deviations (SD). We applied the chi-squared test to compare sociodemographic factors, medical and dental histories, and appointment history components among various compliance status groups (agreed and attended, agreed but failed to attend, refused to attend, could not be contacted). Moreover, we evaluated the associations between certain numerical factors such as age, appointment non-attendance rate, average annual appointment count, number of missing teeth, number of pontics, and compliance status using multinomial logistic regression. A p-value < 0.05 was considered statistically significant.

## RESULTS

### Patient Compliance with Follow-up Atttendance

Of the 314 patients, approximately 90 (28.7%) were unreachable for various reasons, such as disconnected or changed mobile numbers, or non-responsiveness to multiple calls.

Of the remaining 224 patients successfully contacted, 75 (23.9%) declined the appointments. Among these, six patients specified that they were currently residing outside Riyadh.

**Fig 1:**
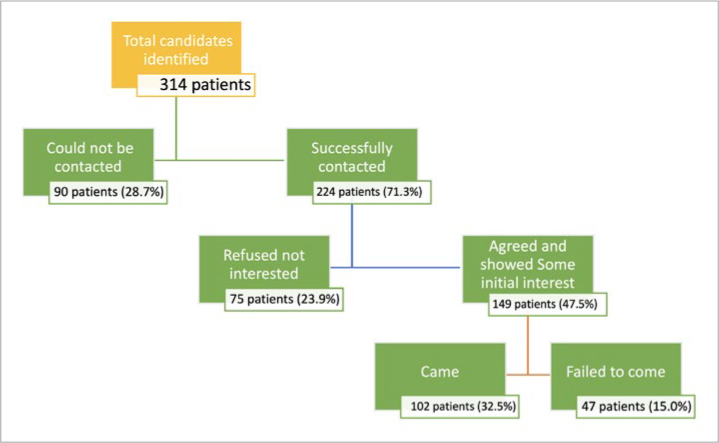
Patients responses to follow-up appointment booking.

Approximately 149 patients (47.5%) displayed some level of interest in attending and participating in the study. Nevertheless, only 102 patients actually attended their appointments (32.5%), while 47 individuals (15.0%) did not show up as scheduled (Fig 1). The final patient responses were categorised into four groups: patients who agreed and attended, patients who initially agreed but did not attend, patients who declined owing to a lack of interest, and patients who could not be reached.

### Factors Affecting Patients’ Atttendance Styles

### Demographic and socioeconomic factors

Most of the study population, 247 (78.8%), were female patients. Of these, 126 initially agreed to participate, but only 83 (33.6%) ultimately attended, and 17.4% failed to show up. However, 23 male patients initially consented to participate, but only 19 (28.6%) actually participated, with a 6% non-attendance rate. A statistically significant sex-based difference was observed, with female patients demonstrating greater interest and compliance in maintaining their appointments (Table 1).

**Table 1:** Sociodemographic data of patients and their responses to attendance (N = 314)

Categories	Groups	No. of patients who attended (percentage%)	No. of patients who failed to attend (percentage%)	No. of patients who were not interested (percentage%)	No. of patients who could not be contacted (percentage%)	Total no. (percentage%)	p-value
**Sociodemographic data**							
Gender	Male	19 (28.4)	4 (6.0)	19 (28.4)	25 (37.3)	67 (21.3)	p < 0.05*
	Female	83 (33.6)	43 (17.4)	56 (22.7)	65 (26.3)	247(78.8)	
Nationality	Non–Saudi	9 (19.6)	8 (17.4)	4 (8.7)	25 (54.3)	46 (14.6)	p < 0.001*
	Saudi	93 (34.7)	39 (14.6)	71 (26.5)	65 (24.3)	268 (85.4)	
Age	< 25	10 (45.5)	7 (31.8)	3 (13.6)	2 (9.1)	22 (7.0)	p > 0.05
	25–34	21 (26.9)	13 (16.7)	21 (26.9)	23 (29.5)	78 (24.8)	
	35–44	29 (30.9)	11 (11.7)2	26 (27.7)	28 (29.8)	94 (29.9)	
	45–54	27 (38.6)	11 (15.7)	11 (15.7)	21 (30)	70 (22.3)	
	55–64	13 (34.2)	5 (13.2)	10 (26.3)	10 (26.3)	38 (12.1)	
	> 65	2 (16.7)	0 (0.0)	4 (33.3)	6 (50)	12 (3.8)	
Marital status	Single	14 (12.7)	22 (20)	44 (40)	30 (27.3)	110 (35.0)	p < 0.001*
	Married	87 (45.8)	25 (13.2)	27 (14.2)	51 (26.8)	190 (60.5)	
	Not indicated	1 (7.1)	0 (0.0)	4 (28.6)	9 (64.3)	14 (4.5)	
Education	High school or less	20 (23.8)	17 (20.2)	20 (23.8)	27(32.1)	84 (26.8)	p > 0.05
	University degree	47 (38.5)	12 (9.8)	27 (22.1)	36 (29.5)	122 (38.9)	
	Postgraduate degree	11 (37.9)	6 (20.7)	5 (17.2)	7 (24.1)	29 (9.2)	
	Not indicated	24 (30.4)	12 (15.2)	23 (29.1)	20 (25.3)	70 (25.2)	
Occupation	Not employed	14 (19.7)	12 (16.9)	20 (28.2)	25 (35.2)	71 (22.6)	p > 0.05
	Employed	59 (38.8)	21 (13.8)	33 (21.7)	39 (25.7)	152 (48.4)	
	Not indicated	29 (31.9)	14 (15.4)	22 (24.2)	26 (28.6)	91 (29.0)	
Area of current residency	Within short distance	26 (34.7)	12 (16.0)	11 (14.7)	26 (34.7)	75 (23.9)	p > 0.05
	Within long distance	48 (32.7)	21 (14.3)	38 (25.9)	40 (27.2)	147 (46.8)	
	Not indicated	28 (30.4)	14 (15.2)	26 (28.3)	24 (26.1)	92 (29.3)	
**Dental history**							
Missing teeth	No	37 (30.3)	22 (18.0)	27 (22.95)	35(28.7)	122 (38.9)	p > 0.5
	Yes	65 (33.9)	25 (13.0)	47 (24.5)	55 (28.6)	192 (61.1)	
Orthodontic treatment	No	95 (32.4)	43.(14.7)	71 (24.2)	84 (28.7)	293 (93.3)	p > 0.5
	Yes	7 (33.3)	4 (19.0)	4 (19.0)	6 (28.6)	21 (6.7)	
Teeth replacement using dental implants	No	77 (30.8)	38 (15.2)	66 (26.4)	69 (27.6)	250 (79.6)	p > 0.5
	Yes	25 (39.1)	9 (14.1)	9 (14.1)	21 (32.8)	64 (20.4)	
**Appointment History**							
Compliance according to percentage	< 30% (compliant)	86 (33.2)	35 (13.5)	57 (22.0)	81 (31.3)	259 (82.48)	p > 0.05
	30–60% (erratic compliance)	15 (28.8)	11 (21.2)	18 (34.6)	8 (15.4)	52 (16.6)	
	> 60 % (non–compliant)	1 (33.3)	1 (33.3)	0 (0.0)	1 (33.3)	3 (1)	
Patient activity status based on date of last appointment	Active (attended within < 3 years ago)	92 (45.3)	34 (16.7)	42 (20.7)	35 (17.2)	203 (64.6)	p < 0.001*
	Moderately active (attended within 3–5 year ago)	5 (7.6)	10 (15.2)	20 (30.3)	31 (47)	66 (21)	
	Non–active patient (attended > 5 years ago)	5 (11.1)	3 (6.7)	13 (28.9)	24 (53.3)	45 (14.3)	

*Chi–squared test indicated statistical significance, p < 0.05.

Most of the sample comprised Saudi patients, accounting for 85.4%, whereas non-Saudi patients made up only 14.6%. Over half of the non-Saudi patients (54.3%, 25 patients) could not be contacted, whereas only 19.6% (or nine patients) attended. Generally, Saudi patients displayed a significantly greater interest in and compliance with appointment attendance (p < 0.001).

The patients included in the study had an age range of 18–70 years, with a mean age of 41.1 ± 12.1 (Table 3). Upon categorising the patients by age, it was observed that younger patients (under 25 years) attended their appointments more often than all other age groups, whereas the eldest group ( > 65 years) attended the least. Remarkably, half of the oldest age group could not be contacted, contrary to the mere 9.1% contact failure rate in the youngest age group. Atttendance patterns and interest levels were similar across the remaining age groups, with the overall differences among them proving statistically insignificant (Table 1).

Of the sample, approximately 190 patients (60.5%) were married, and 110 (35%) were single. Interestingly, 4.5% of the patients chose not to disclose their marital status. Intergroup comparisons revealed statistically significant differences among the groups. Married patients demonstrated higher interest in appointments, attended appointments more consistently, and had the lowest number of missed appointments (Table 1).

We assessed the effect of family size on patient compliance. However, only a small portion of patients (130 or 41.4% of the sample) provided information on their total family members, which varied from 2 to 13, with an average of 6.38 ± 2.49 (Table 3). While a trend was observed where individuals from smaller families seemed more likely to attend appointments, the relationship was not statistically significant (p > 0.05).

Evaluation of the patients’ educational levels, occupations, and current residencies showed numerical differences, but statistical analysis did not find statistically significant associations with their attendance patterns. Notably, approximately half of the sample was missing at least one of these data points (not indicated) (Table 1). We further analysed these factors in patients with complete datasets (n = 148). After filtering patient data and removing those with missing or non-indicated information, only employment status showed a statistically significant association; employed patients demonstrated better attendance and fewer instances of non-attendance (p < 0.05) (Table 2).

**Table 2:** Sociodemographic data of patients and their responses to attendance after removal of patients with missing data (N = 148)

Categories	Groups	No. of patients who attended (percentage%)	No. of patients who failed to attend (percentage%)	No. of patients who were not interested (percentage%)	No. of patients who could not be contacted (percentage%)	Total no. (percentage%)	p-value
**Sociodemographic data**							
Marital Status	Single	7 (11.5)	11 (18.0)	24 (39.3)	19 (31.1)	61 (41.2)	p < 0.001*
	Married	43 (49.4)	13 (14.9)	6 (6.9%	25 (28.7)	87 (58.8)	
Education	Highschool or less	12 (23.1)	13 (25.0)	8 (15.4)	19 (36.5)	52 (35.1)	P > 0.05
	University degree	28 (36.4)	10 (13.0)	19 (24.7)	20 (26.0)	99 (52.0)	
	Postgraduate degree	10 (52.6)	1 (5.3)	3 (15.8)	5 (26.3)	19 (12.8)	
Occupation	Not employed	9 (18.8)	9 (18.8)	9 (18.8)	21 (43.8)	48 (32.4)	p < 0.05*
	Employed	41 (41.0)	15 (15.0)	21 (21.0)	23 (23.0)	100 (67.6)	
Area of current residency	Within short distance	16 (31.4)	9 (17.6)	8 (15.7)	18 (35.3)	51 (34.5)	P > 0.05
	Within long distance	34 (35.1)	15 (15.5)	22 (22.7)	26 (26.8)	97 (65.5)	

*Chi square test indicated statistical significance, p < 0.05

**Table 3:** Descriptive statistics of demographic factors, dental history and appointment history elements

Item	Range	Mean ± SD
Age	18–70	41.1 ± 12.1
Total family members	2–13	6.38 ± 2.49
No. of missing teeth	1–15	2.42 ± 3.01
No. of implants per patient	1–7	0.46 ± 1.16
No. of pontics per patient	1–6	0.93 ± 7.91
Total number of appointments given for the patient	1–176	53.69 ± 34.4
Duration of treatment (in years)	1–10	4.93 ± 2.59
Average number of appointments per year	1–99	14.4 ± 11.6
Number of missing appointments	0–62	10.98 ± 11.1
Percentage of missing appointments	0–87.5	19.1 ± 13.3
Duration since date of last appointment (in years)	1–10	2.12 ± 2.49

### Medical history

The review of medical histories showed that the physical status of 45.9% of the sample was classified as ASA I, 47.8% as ASA II, 4.5% as ASA III, and 0.3% as ASA IV. Nonetheless, there was no recorded medical history in 1.6% of the patients. These participants made very few visits to the collage (around three), and none could be contacted.

**Fig 2:**
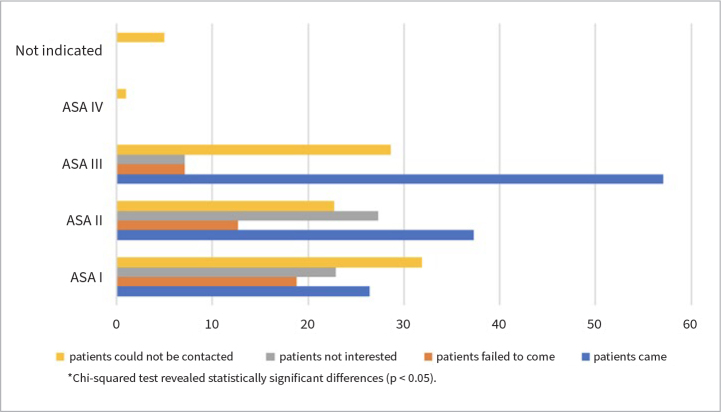
Medical history categories and attendance patterns.

Analysis showed that over half of the patients with ASA class III (57.1%) attended their appointments. This group also had the lowest non-attendance rate (7.1%). A subgroup comparison revealed a statistically significant difference between the groups (Fig 2).

Interestingly, the group with the highest percentage of patients who were disinterested in their appointments belonged to the ASA II. Further analysis revealed that this trend was particularly prominent in the subgroup of patients with multiple controlled systemic diseases (three or more).

### Dental history

Overall, 122 patients (38.9%) had all of their teeth present, whereas 192 patients (61.1%) had lost at least one tooth. Patients with missing teeth attended appointments slightly more frequently, and missed fewer appointments. However, the chi-squared test revealed that these differences were not statistically significant (Table 1). Further analysis found the number of missing teeth per patient ranged from 1 to 15, with a mean of 2.42 ± 3.01 (Table 3). Nonetheless, no statistically significant association was identified between the number of missing teeth and the patients’ tendency to attend or miss appointments (p > 0.05) (Table 4).

**Table 4:** Multinomial logistic regression between age, dental history, appointment history and attendance pattern (N=314)

Factors		Adjusted associations	
		OR [95%CI]	p-value
**Age**			
	Agreed to attend	1.00 [Reference]	
	Failed to attend	0.97 [0.93-1.00]	0.070
	Refused to attend	1.02 [0.99-1.05]	0.308
	Could not be contacted	1.01 [0.99-1.04]	0.328
**No of missing appointments**			
	Agreed to attend	1.00 [Reference]	
	Failed to attend	0.96 [0.92-1.00]	0.056
	Refused to attend	0.97 [0.93-1.00]	0.070
	Could not be contacted	0.94 [0.90-0.98]	0.001*
**Percentage of missing appointments**			
	Agreed to attend	1.00 [Reference]	
	Failed to attend	1.05 [1.01-1.08]	0.012*
	Refused to attend	1.03 [0.99-1.06]	0.146
	Could not be contacted	1.03 [0.99-1.06]	0.118
**Average no of appointments /year**			
	Agreed to attend	1.00 [Reference]	
	Failed to attend	1.06 [1.01-1.11]	0.011*
	Refused to attend	1.08 [1.04-1.13]	0.000*
	Could not be contacted	1.07 [1.03-1.12]	0.001*
**No. of missing teeth**			
	Agreed to attend	1.00 [Reference]	
	Failed to attend	1.07 [0.93-1.23]	0.348
	Refused to attend	0.93 [0.82-1.05]	0.232
	Could not be contacted	0.96 [0.86-1.07]	0.467
**No. of replaced teeth by pontics**			
	Agreed to attend	1.00 [Reference]	
	Failed to attend	0.95 [0.63-1.44]	0.812
	Refused to attend	1.03 [0.96-1.11]	0.452
	Could not be contacted	1.03 [0.96-1.11]	0.427

*Statistically significant, p < 0.05.

**Fig 3:**
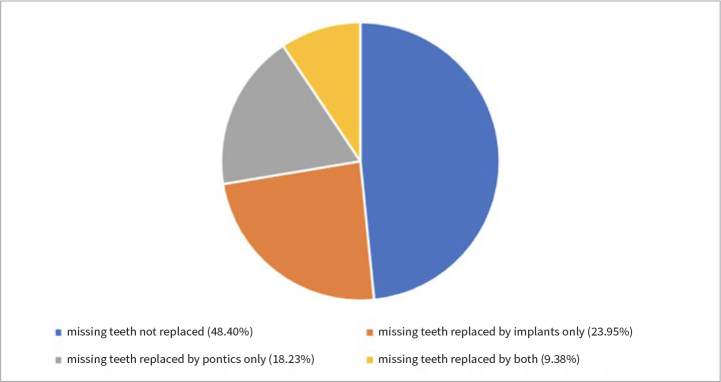
Distribution of missing teeth according to their replacement status.

Only 99 patients (51.6% of those with missing teeth) had some or all of those teeth replaced, whether with implants or fixed prosthodontics. Specifically, 64 patients (20.4% of the sample) had at least one dental implant, with the number of implants ranging from 1 to 7 per patient, and an average of 0.46 ± 1.16 (Fig 3; Table 3). Overall, those with dental implants appeared to be more interested in and compliant with their appointments. Nevertheless, there appeared to be no significant difference in patient attendance patterns based on their tooth replacement status (presence of implants or pontics) (p > 0.05) (Tables 1 and 4).

Finally, approximately 21 patients (6.7%) had a history of orthodontic treatment. However, this history did not seem to statistically significantly affect compliance rates (Table 1).

### Appointment history

Overall, the patients received treatment at the college with considerable variation in their range of services use. The number of treatment appointments varied widely, ranging from 1 to 176, with a mean of 53.7 appointments over periods of 1–10 years. On average, the patients had approximately 14.4 appointments per year (Table 3). Further analysis showed a statistically significant positive correlation between the number of annual visits and patients’ attendance at follow-up appointments offered in this study (p < 0.05, Table 4).

The number of missed appointments varied from 0 to 62 (mean: 10.98 ± 11.1), and the corresponding percentages ranged from 0 to 87.5%, averaging 19.1% (Table 3). A statistically significant relationship was found between the percentage of missed appointments and the patient compliance rate (Table 4). However, when the patients were categorised into three general compliance groups based on the percentage of missed appointments, a comparison between their original and current attendance patterns showed no statistically significant difference (Table 1).

Patients were categorised based on their most recent appointment date at the college: active patients (64.6%), moderately active (21%), and non-active patients (14.3%). Most non-active patients (53.3%) were unreachable. Conversely, active patients were easily contacted, most likely to attend appointments, and had the best attendance rate for current appointments. These differences were statistically significant (p < 0.001).

## DISCUSSION

Effective service utilisation and efficient management of time and resources require patients to attend scheduled appointments.^17^^,^^25^ However, research has indicated disparities in appointment attendance rates depending on the nature of the appointment (such as consultation, pain management, follow-up, and participation in research^54^^,^^59^) and patient category (new vs regular;^17^^,^^57^ self-referred vs referred by others^37^).

This study aimed to evaluate the patients’ willingness to attend follow-up and maintenance appointments for periodontal care after crown-lengthening surgery. Crown lengthening is a surgical procedure often performed pre-prosthetically to preserve compromised teeth.^31^ Prior studies have suggested that patients who have received surgical periodontal treatments are observed to follow up on dental appointments more diligently.^11^^,^^27^^,^^52^^,^^60^ This may be attributed to their heightened understanding of complex treatment plans and their eagerness to circumvent additional surgeries.^37^

Multiple studies have shown diverse compliance rates for dental follow-ups and periodontal maintenance therapies,^33^^,^^35^^,^^37^^,^^41^^,^^47^^,^^60^ ranging between 16%^60^ and 54%.^41^^,^^47^ In our study, approximately one-third of patients successfully attended follow-up appointments. Consistent with this, Miyamoto’s report indicating compliance rates to fluctuate between 32.5% and 35.6%,^35^ which aligns with the findings of Mendoza et al.^33^ Notably, various authors employed different classification systems to evaluate compliance rates based on the number or percentage of missed appointments during maintenance periods.^20^ This inconsistency in categorisation may have contributed to the observed variation in the results.

Several patients were not interested in the offered visit. This could stem from multiple factors, including the perception that further treatment is not needed,^20^ and the tendency to undervalue the importance of follow-up dental appointments.^50^

### Sociodemographic Factors

Sociodemographic factors include age, gender, race as well as multiple measures of socioeconomic status.^57^^,^^59^ Socioeconomic status is a broad term that refers to the capacity of an individual, family, or area to produce or use significant societal resources.^34^ Evaluating a patient’s socioeconomic status is often challenging and requires a meticulous appraisal of diverse factors. Non-responses to certain variables, especially those associated with income,^51^ serve as key limitations to socioeconomic measurements. In Saudi society, various social hurdles can cause individuals to feel reluctant to share this information. They may feel uncomfortable revealing specifics about their education, occupation, marital status, or number of children. Additionally, the lack of understanding about the importance of this data for healthcare contribute to the large amount of unreported data.

Our sample had a higher proportion of female patients primarily because the initial data gathered for patients who underwent crown-lengthening surgeries included more female patients. This indicates that women may prefer preserving their natural teeth more often than extraction or dental implant placement, a choice seemingly favoured by men.

Our study highlighted significant sex-related differences. Females showed more interest in appointment attendance, and subsequently attended appointments more frequently. This aligns with existing literature, which frequently indicates that female patients demonstrate a greater commitment to attending appointments and health maintenance^30^ compared to males.^9^ Interestingly, however, there is conflicting evidence. Perrell-Jones and Ireland^45^ have indicated that males can often be more compliant than females, while similar compliance trends across genders have been noted in other societies as well.^37^^,^^56^

Our college serves patients of many nationalities across a range of specialties; however, our sample predominantly comprises residents of the host country. This suggests that local citizens are highly aware of the superior quality of the services provided by our institution. Notably, reaching out to most foreigners who may have already left the country presents challenges. Additionally, our data showed higher instances of missed appointments in foreign patient demographics. This could be explained by their work commitment and preferences for evening appointments. Gatrad^24^ identified a similar trend in the UK, with English patients showing a higher compliance rate than non-citizen Asians, a finding attributed to language barriers.

Ojima et al^42^ discovered that age greatly affects patient compliance, with older individuals being more inclined to keep appointments. This trend is consistent with the findings of other studies.^12^^,^^45^ However, this contradicts reports of lower compliance among older patients from some other studies.^4^^,^^37^ Davies et al^17^ notably found that non-attendance rates fall with age until reaching the 75- to 79-year-old bracket; at this point, they begin to rise. Although our study recognised slight differences between the youngest and oldest age groups, we found them to be statistically insignificant and irrelevant. A similar pattern was observed in a separate domestic study.^6^

Interestingly, some authors have not found statistically significant associations between individual sociodemographic factors and compliance. Nevertheless, some statistical significance was observed when examining the collective impact of these factors.^40^ For instance, a combined analysis of gender and age by Davies et al^17^ and his team revealed that males were more likely to fail to comply until the age of 65 years, after which both sexes shared similar rates.

Our study reveals a notable disparity in appointment attendance rates between single and married individuals. This discovery aligns with the research conducted by Daggy et al^14^ and Ranjan et al.^46^ Nonetheless, this conflicts with Barakati’s findings,^5^ which suggested that married females, particularly homemakers, constituted the majority of absentees.

Our findings indicate that members from larger families are slightly more inclined to miss appointments irrespective of their marital status, although the difference is statistically insignificant. This observation implies that senior individuals in these families face difficulties in ensuring sufficient care for each member, leading to less attention to scheduled follow-ups. Corroborating this, Gatrad^24^ mentioned that patients responsible for arranging childcare services are more prone to missing consultations, particularly in families with younger children. Conversely, Ranjan et al^46^ stated that the larger the number of dependents in the family, the more interest there is in participating in health-related studies, possibly driven by anticipated financial rewards.

Our findings did not reveal any notable correlation between the patients’ education or occupation levels and their adherence to scheduled appointments. Furthermore, relevance emerged only after excluding patients with incomplete information, revealing that employed patients were more likely to attend appointments. Similarly, Patel et al^43^ found that most patients who missed dental treatment appointments were unemployed.

Daggy et al^14^ discovered a statistically significant relationship between longer travel distances and compliance levels. Despite the minor differences reported concerning patient residency areas in our study, we did not find a prominent relationship, except for those living outside the city. This could be attributed to the College of Dentistry offering free, high-quality care that is easily accessible, thereby attracting patients from distant areas. Furthermore, a patient’s residential location may not be the sole factor affecting attendance. For instance, those who study, work, or have family members working near the university may find it easier to attend regular dental appointments. This aligns with other studies in which location did not statistically significantly impact compliance levels.^33^^,^^42^

### Medical History

Patient medical histories statistically significantly affected compliance rates. Patients in ASA III category were more compliant, whereas those in the ASA I category showed the highest rates of missed appointments and the least interest in attending.

The literature reveals a specific correlation between certain systemic diseases and patient no-show rates. For instance, Daggy et al^14^ noted that conditions such as major depression and drug dependence, had a higher association with missed appointments. Conversely, patients with cardiac disease, diabetes, or chronic obstructive pulmonary disease were more likely to comply with their appointments.^14^

Patients with mild and well-managed systemic diseases usually have no difficulty performing dental procedures. However, those with multiple systemic diseases and various treatments might need to expend additional effort to adequately control their health and comply with each medication regimen. This increased effort could also extend the time spent visiting doctors and scheduling appointments. Such circumstances may account for our findings, which indicate a reduced inclination of these patients to attend dental follow-up appointments.

### Dental History

The evaluation of the number of previously lost teeth showed no statistically significant impact on patient compliance, corroborating the findings of other studies.^33^^,^^35^ However, it was observed that patients anticipating poor dental outcomes were often less compliant than those expecting better outcomes. This reduced compliance may stem from the fear of further tooth loss.^60^

Previous studies have explored the effect of a history of dental implant procedures on patient adherence to supportive periodontal therapy.^11^^,^^27^ The higher compliance rate among these patients could be attributed to the cost of the procedure, which motivated them to maintain better dental care. However, our study did not identify a statistically significant correlation between dental implant history and attendance at follow-up visits. One possible explanation could be that the implants were not necessarily placed at the same institution or by the same periodontist. Furthermore, the specific characteristics of the participants may have affected this study. All the participants in this study underwent a periodontal surgical procedure, specifically crown lengthening.

Interestingly, the history of orthodontic treatment had no statistically significant effect on appointment adherence. This may be because some of these patients were already scheduled for regular visits by their orthodontists, eliminating the need for follow-up with other specialists.

### Appointment History

The history of the patients’ previous appointments was examined. The average number of annual dental appointments was directly correlated with patient commitment to follow-up visits. This may be due to the fact that regular appointments could signal a complex treatment plan, increasing patient awareness and adherence to appointments. Daggy et al^14^ observed that a greater number of scheduled appointments resulted in fewer non-attenders.

A larger number of appointments corresponds to shorter intervals between appointments. The impact of such intervals has been assessed previously with varying results. Some studies found no statistically significant relation,^5^ while others revealed a direct proportionality with patient compliance.^30^ This contrast can be attributed to the patients’ commitment to and understanding of their oral health status. However, when it comes to periodontal maintenance, traditional studies consistently report a decrease in compliance with an increase in the number of scheduled visits per year.^21^^,^^60^

Studies have also shown decreased adherence in patients with fewer than four appointments.^14^ Similarly, in our study, this group was the least interested in attending follow-up visits and was the most challenging to reach.

Our findings also showed that the time since a patient’s last appointment was statistically significantly associated with their likelihood of attending and that older patients were the hardest to reach and the least interested in attending. This could be due to changes in various factors surrounding these patients, such as sociodemographic conditions causing them potentially to visit other dental clinics. Similarly, Davies et al^17^ observed a statistically significant difference in the non-attendance rates between new and established patients, with new patients missing more appointments. In their study, new patients were defined as those with no history of appointments in the previous year.^17^ Numerous authors have noted that patient compliance with supportive therapy tends to decline over time.^17^^-^^19^

Several studies have identified the patients’ prior rates of missed appointments as statistically significant factors in their future attendance, indicating a correlation with diminished compliance.^6^^,^^14^ Similarly, our study found a direct link between a patient’s history of non-attendance and the likelihood of not showing up for follow-up appointments.

Adherence to follow-up visits is crucial. Therefore, research on patient attendance patterns and barriers is vital. The findings of these studies should inform strategies aimed at enhancing patient attendance at these visits.^49^ Measures such as implementing a reminder system that incorporates automated text messages, e-mails, or calls, are recommended.^1^^,^^53^ In addition, offering flexible scheduling options for various times and days can accommodate busy lifestyles.^28^ Moreover, proper patient education that conveys the importance of regular periodontal follow-up in maintaining oral health and outlines the potential consequences of missed appointments, patient understanding, and commitment can be reinforced.^15^^,^^32^

Despite the findings of this study, it has several limitations. The principal limitation was that the sampling technique involved only a specific patient group (those with a history of crown-lengthening surgery) and represented a single point in time. In fact, having research as part of broader research may limit patient selection and affect the findings. Another limitation was the sourcing of sociodemographic data from files, as these data are typically updated only for compliant patients and those with recent college visits. Notably, evaluating patients with poor adherence was a challenge in most cited studies, potentially making their data less precise. Unlike in many other research scenarios, no financial or special dental incentives were provided to patients beyond those offered during regular maintenance visits, a factor that may have encouraged patient attendance and participation. Additionally, the study setting likely mirrors certain aspects of different studies related to appointment attendance, periodontal maintenance, and patient participation in research. Notably, our institution continues to struggle to provide consistent periodontal maintenance services to all patients. This challenge is primarily due to clinicians’ focus (mainly dental students) on regular patient treatments, and patients’ limited understanding or reluctance in terms of attending follow-up visits. Therefore, integrating follow-up appointments with other investigations is crucial to deliver maintenance care.

## CONCLUSION

Despite the reported limitations, our study showed an overall compliance rate comparable to the averages reported in other studies. Different factors influence these rates to varying extents, with patterns that are often unique to this specific population. These findings prove that compliance is a complex multifactorial issue. Owing to the importance of dental follow-up appointments, there is a need for more controlled studies to examine appointment-keeping behaviors and their influencing factors, comparing them to worldwide norms. These studies are crucial for the selection and implementation of the most suitable techniques to motivate patients to commit to these visits.
